# Primary and Secondary Emissions Reduction Using Cylinder Deactivation Strategies for Gasoline Direct Injection Engines in Hybrid Vehicles

**DOI:** 10.1007/s42154-024-00328-6

**Published:** 2025-04-10

**Authors:** George Brinklow, Jose Martin Herreros, Soheil Zeraati-Rezaei, Athanasios Tsolakis, Paul Millington, Amy Kolpin

**Affiliations:** 1 Department of Mechanical Engineering, School of Engineering, University of Birmingham, Edgbaston, Birmingham, B15 2TT UK; 2 Johnson Matthey Technology Centre, Blount’s Court, Sonning Common, Reading, RD4 9NH UK

**Keywords:** Three-way catalyst, Catalyst thermal behavior, Pollutant emissions, Unregulated emissions, Hybrid vehicle, Cylinder deactivation

## Abstract

Stricter CO_2_ and local air quality targets are increasing the demand for electrified powertrains including hybrid electric vehicles (HEVs). The impact of an electrified vehicle powertrain on the catalytic performance of the emissions control system presents the challenge of multiple cold/warm starts during vehicle operation. This work investigates advanced energy efficient technologies to understand and enhance the catalytic reduction of primary and secondary emissions under challenging HEV operation conditions. A novel strategy based on the concept of engine cylinder deactivation is experimentally studied at various starting catalyst temperatures aiming to reduce the time required to reach catalyst light-off temperature and thus tailpipe emissions. Unregulated secondary emissions (NH_3_ and N_2_O) are also investigated, which are expected to become more pertinent in the future. This work demonstrates that operating under the studied strategy for a short period of time increased TWC temperature by up to 300 °C and reduced mass-based emissions of CO, NO, HCs, N_2_O and NH_3_. These findings are significant to inform the optimization of energy efficient catalyst heating strategies for HEVs in order to reduce both primary and secondary emissions.

## Introduction

Whilst in the future it is expected that carbon-free/carbon–neutral fuels will become available, even optimistic projections expect that fossil fuel internal combustion engine (ICE) powered transportation will be widely used in the future [1]. One mid-term solution to reduce CO_2_ emissions and increase energy efficiency in the transportation sector has been the uptake of hybrid electric vehicles (HEVs) [2]. These powertrains combine two or more different propulsion systems. In most cases, it is an ICE with an electric motor that will reduce CO_2_ emissions in comparison to conventional ICE vehicles [3, 4].

Whilst HEVs show promise for reducing CO_2_ emissions in the mid-term, the intermittent operation of the ICE within an HEV produces a challenging environment for the three-way catalyst (TWC). The TWC can successfully convert pollutant species, but only at temperatures higher than the light-off temperature (typically above 250 °C) and at lambda values around stoichiometric [5, 6]. Therefore, emissions of CO, hydrocarbons (HCs) and NO_*X*_ could slip past the TWC if it is allowed to cool below the light-off temperature whilst the engine is off [7]. During HEV operation there are multiple start events where catalyst performance will be inhibited [8].

There have been several studies that have assessed the performance of the TWC to abate primary emissions in a HEV and further studies that have evaluated different TWC heating strategies. Traditionally, rapid TWC light-off is achieved through delayed spark timing [9]. However, it has a thermal efficiency penalty associated with it due to the increased heat loss through the exhaust. There are studies that have specifically improved TWC performance in HEVs with the adjustment of the control/energy management strategy (EMS). Michel et al. demonstrated improved TWC performance in a HEV leading to a reduction in CO and NO_*X*_ of 30% and 10%, respectively, through applying a “smart catalyst heating” strategy that focused on minimizing pollutant species. However, it did carry a fuel consumption increase [10]. Giles des Buttes et al. also studied how changing the EMS from a fuel consumption centered strategy to a pollutants centered strategy reduced emissions of CO, NO_*X*_ and HCs by 39%, 15% and 34%, respectively. It did also result in a fuel consumption increase of 0.5% [11]. Benegiamo et al. also investigated the effect the EMS of a HEV had on the emissions performance. This differed from the work by Michel et al. and Giles des Buttes et al. as it focused on the catalyst temperature and warmup period to reduce the time the catalyst spent below light-off temperature. The catalyst heating time was reduced but with a 2% increase in fuel consumption [12].

There has also been research into other catalyst heating strategies which include secondary air injection and cylinder deactivation (CDA). Secondary air injection before the TWC can be used to generate exothermic reactions between the injected air and combustible species in the exhaust. This was investigated by Lee and Heywood, where reductions in HC and CO emissions of 46%–88% and 37%–93%, respectively, over the first 25 s from the start-up were achieved [13]. The variation depended upon the different lambda and air injection flow rates in addition to the effect of spark retardation. Smith et al., Sabu et al. and Borland et al. all found similar increases in catalyst temperature of around 150 °C–200 °C with the adoption of secondary air injection [14–16].

CDA has also been studied as a method for heating the TWC. Kumar et al. witnessed an increase in exhaust gas temperature of 130 °C when applying a CDA strategy. However, due to the reduced exhaust flow rate caused by deactivation of the valves it was studied no further [17]. Luo et al. studied the effect of CDA further by looking at dynamic skip fire (DSF). In this case, the activated and deactivated cylinders vary from cycle to cycle. This method successfully increased catalyst temperature by approximately 100 °C with an additional investigation using a deactivated cylinder to pump air increasing the catalyst temperature by approximately 150 °C [17]. Millo et al. investigated CDA as a means to improve efficiency, but also reported an increase in TWC temperature due to the oxidation of fuel species present in the exhaust because of the enrichment of the firing cylinders [18]. However, the effect of this at varying catalyst start temperatures and upon unregulated emissions was not addressed.

Whilst there are studies investigating CDA to reduce the time required to reach TWC light-off temperature, these involve the deactivation of the valves, which increase complexity. CDA with secondary air injection has been also proven for catalyst heating, but through a DSF approach. This work aims to combine secondary air injection and CDA to a powertrain that does not operate with DSF, as a simpler as well as more energy and cost-effective strategy to enhance emissions control. The novelty and significance of this work are further evidenced by providing new knowledge related to the formation of N_2_O and NH_3_ over the TWC as secondary emissions as well as proposing and demonstrating efficient pathways for their control simultaneously to primary emissions. These secondary species are currently not regulated but are becoming the focus of attention due to their respective greenhouse gas emissions potential and contribution to atmospheric particulate matter [19, 20].

## Methodology

The experiments were carried out at the University of Birmingham Engine Test Laboratory using a modern 3-cylinder 1.5 l turbocharged GDI engine. This engine was a contemporary EURO 6 engine that featured multiple friction reducing and lightweight solutions in addition to a modern low platinum group metal (PGM) loading TWC. The TWC was provided by Johnson Matthey PLC with a total PGM loading of 30 g/ft^3^. This was made up of 23 g/ft^3^ of palladium and 7 g/ft^3^ of rhodium.

**Fig. 1:**
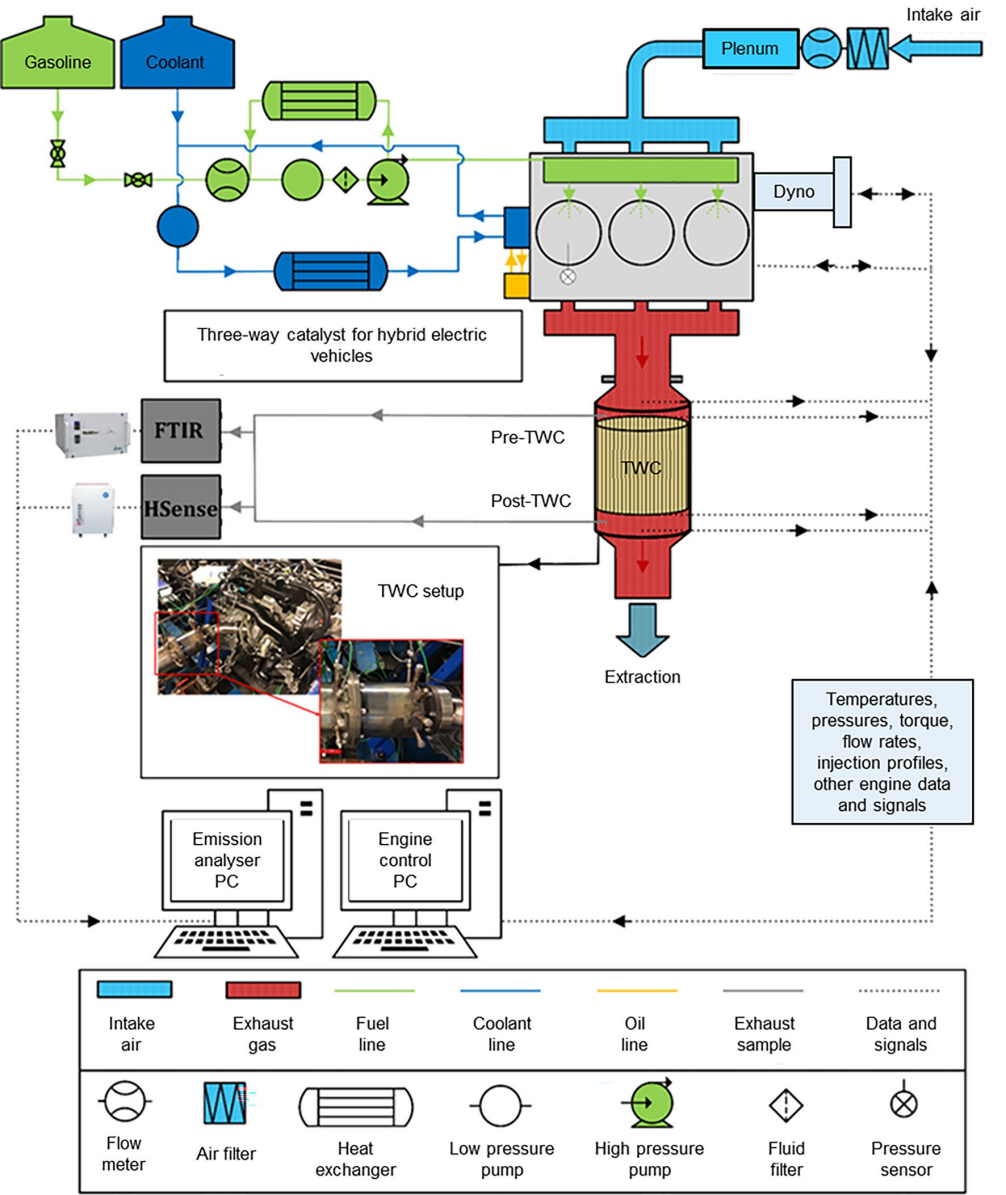
Experimental test set up schematic

The TWC was mounted in a close coupled TWC position and then instrumented with k-type thermocouples to measure the inlet, outlet and brick temperatures. A UEGO sensor was placed before the TWC in order to understand how CDA periods affected the total engine-out lambda. Emissions before and after the TWC were measured with an MKS multigas FTIR emissions analyzer and Hydrogen (H_2_) V&F EIMS-HSense hydrogen analyzer. A full schematic of the experimental setup is shown in Fig. 1.

The engine was started at various temperatures defined by oil, coolant and pre-TWC temperature (Table 1) in order to replicate the conditions that an engine and TWC would be exposed to in a modern HEV. Experiments completed included baseline operation for comparison and CDA to study the effect on TWC thermal management. For the CDA experiments, the timing at which the CDA period started was key as it determined TWC temperature at the beginning of the CDA period (Table 1).

**Table 1:** Engine and TWC temperatures at the start of the experimental sequence and TWC temperature at the start of the CDA period

Experiment number	TWC Temp. (°C)	Oil Temp. (°C)	Coolant Temp. (°C)	TWC Temp. at CDA start (°C)
1	20.8	19.4	19.5	117.5
2	47.1	49.8	46.7	181.8
3	64.5	55.6	54.1	239.9
4	85.2	62.4	61.5	328.3

The novel CDA procedure was implemented by cutting the fuel injection to cylinder 1 whilst the valves for cylinder 1 remained active. It differed from conventional CDA strategies where deactivation of the valves takes place to prevent deterioration in TWC performance. With the valves remaining active, it was possible to use the deactivated cylinder to pump air to the aftertreatment system. Due to this CDA strategy also controlling fuel injection to the firing cylinders, the concentrations of unburnt HCs, CO and H_2_ were increased through overfueling. It then allowed the air pumped by the deactivated cylinder to mix and react with the rich combustion gases and produce heat within the exhaust to reduce the time required to reach TWC light-off temperature.

The novelty of this strategy comes from the amalgamation of the air pumped by the deactivated cylinder and richer combustion gases from the firing cylinders controlled by the fuel injection to achieve a quicker catalyst light-off. By combining the reduced pumping losses of a CDA strategy with the increased exhaust gas temperature of combustion occurring within the exhaust manifold, this proposed strategy is expected to have a reduced fuel consumption penalty compared to other catalyst heating strategies.

**Fig. 2:**
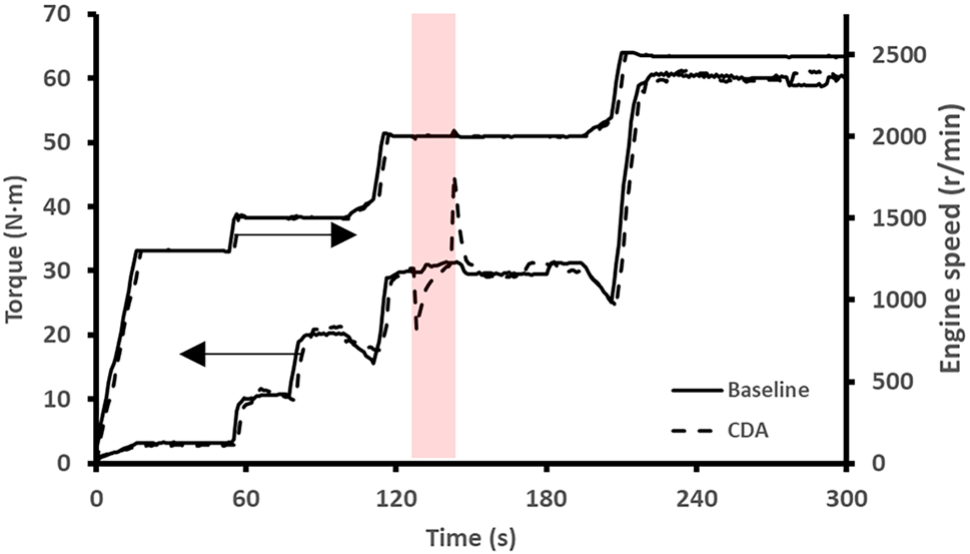
Engine speed and load during the baseline and CDA start-up experimental sequences. The CDA period is shown by the red shaded region

The calculation for TWC light-off time reduction was done by comparing the time taken for the baseline and CDA engine starts to reach approximately > 95% TWC conversion efficiency and then presenting it as the percentage reduction. The start procedure in terms of both engine speed and load is shown in Fig. 2 for the first 5 min of engine operation for both the baseline and CDA experiments. For the CDA experiments (shown by the dashed line), the CDA period lasted for 15 s from 130 to 145 s after the engine started.

The engine speed during the start procedure was not affected, while there was a variation in the torque when the cylinder was deactivated and then reactivated. As the hybridization level of the vehicle powertrain increases, it will become easier for any electrical motors to smooth out these peaks and drops in engine torque during any cylinder deactivation switching events [21]. In addition, the torque variation can also be minimized through optimization of the flywheel and adoption of various levels of torque converter slip [22] or through a more gradual transition [23].

## Results and Discussions

### Effect of Cylinder Deactivation on Catalyst Temperature

**Fig. 3:**
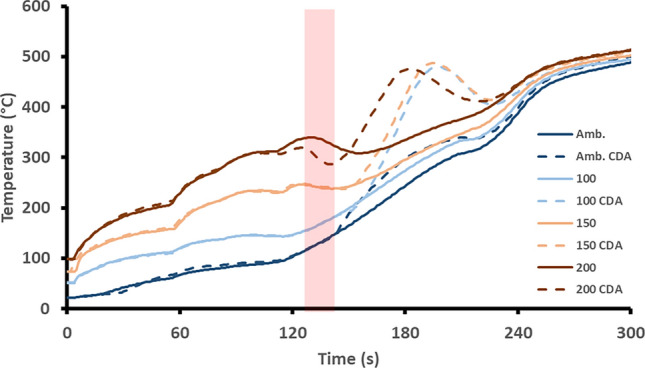
TWC monolith inlet face temperature comparison for each start-up experimental sequence. The CDA period is shown by the red shaded region

The TWC temperature is compared for the baseline and CDA engine start sequences at each of the starting temperatures studied (see Fig. 3). The increase in TWC temperature seen for all CDA periods was related to the oxidation reaction of the excess oxygen from the deactivated cylinder and the CO, H_2_ and HCs from the richer firing cylinders [24, 25].

The highest increase in catalyst temperature (almost 300 °C) was when starting with a TWC temperature of 100 °C and 150 °C. After the maximum increase in temperature was reached, the temperature of the catalyst did reduce again for each CDA strategy, but it did not reduce to less than the catalyst temperature during the baseline experiment. Therefore, showing there to be no negative effects of the CDA period on catalyst temperature. When applying this strategy at higher catalyst starting temperatures (e.g. 200 °C), the increase in catalyst temperature was smaller. For the ambient start, there was a limited increase in catalyst temperature (around 50 °C). This is because this catalyst heating strategy is dependent upon the catalyst temperature being close to—but not at—the light-off temperature, as shown in Table 1. If the temperature is too low, the oxygen from the deactivated cylinder will not react over the catalyst with the excess CO, H_2_ and HCs from the two richer cylinders [26]. This finding is significant as it evidences that there is an optimal temperature range to which this strategy is applicable. It then could be used to flag when operating in a CDA mode would enhance TWC warm-up.

### Effect of Cylinder Deactivation on Regulated Emissions

**Fig. 4:**
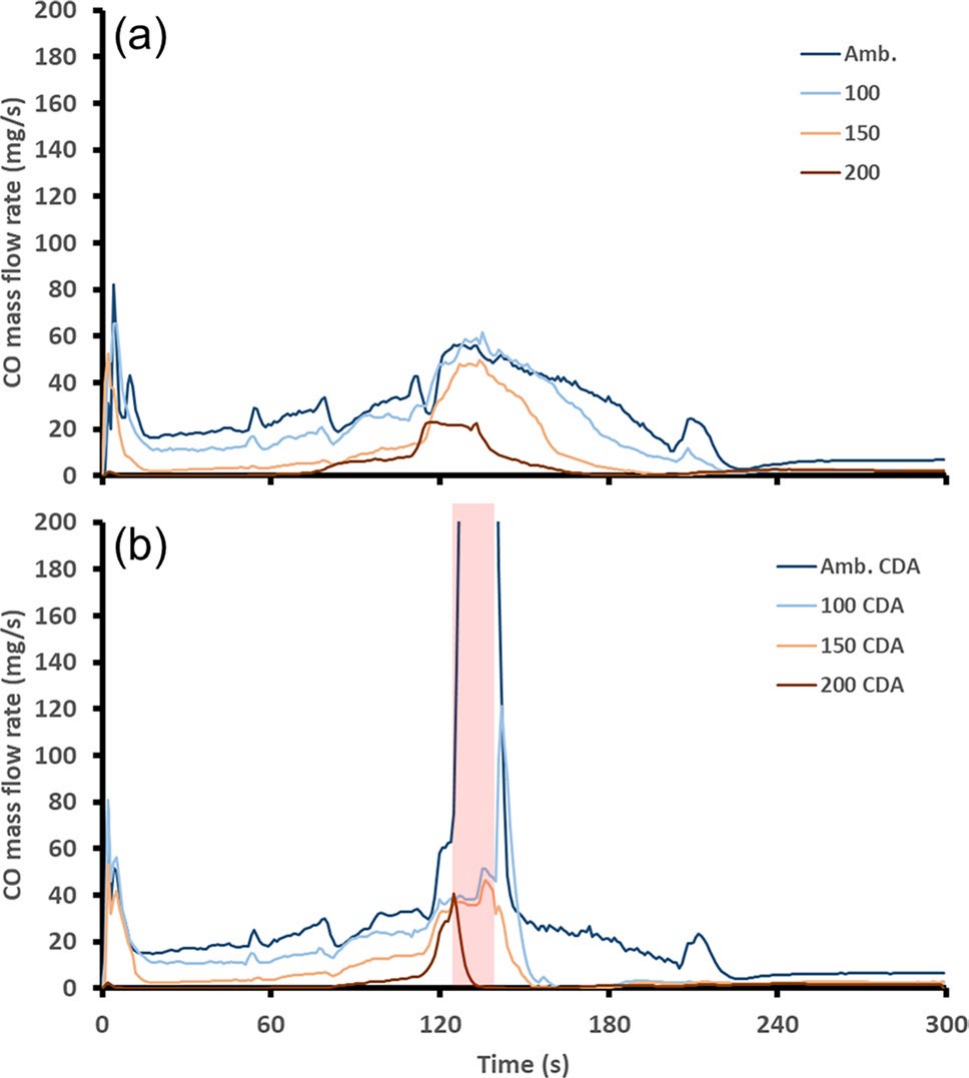
Post-TWC CO mass flow rate, **a** shows the baseline, **b** shows the start-up featuring the 15-second CDA period shown by the red shaded region

**Fig. 5:**
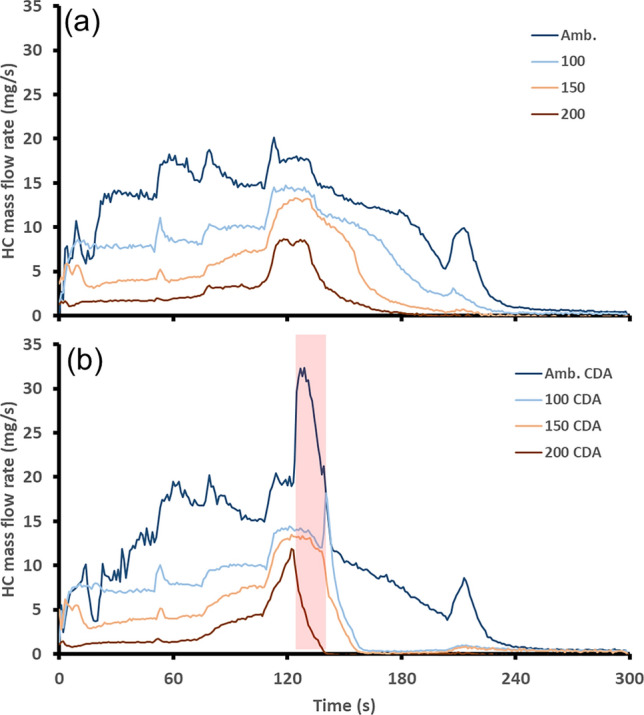
Post-TWC HC mass flow rate, **a** shows the baseline, **b** shows the start-up featuring the 15-second CDA period shown by the red shaded region

Emissions of CO, NO and HC are all regulated by the current emissions standards and therefore are presented here. The mass flow rate of CO downstream of the catalyst is shown in Fig. 4(a) for the baseline engine starts. For these experiments, there was a reduction in the time taken for the TWC to reach complete conversion as the starting temperature of the catalyst increased. This was due to the TWC starting from a higher temperature. What was also observed was the different CO mass flow rates during the first part of the experiment before the TWC reached light-off temperature. For the engine starts at TWC temperatures at 150 °C or greater, there was almost no slip of CO from the catalyst. This resulted from not only the TWC operating at a higher temperature, but also the improved combustion efficiency of a warmer engine compared to a cold one. Therefore, the higher temperature starts with a higher combustion efficiency leading to lower engine out/pre-TWC CO emissions [27]. This reduced the demand on the TWC and therefore lowered CO mass flow rates downstream of the TWC. The same trend was also observed in the baseline results for the downstream HC mass flow rate presented in Fig. 5(a) with the HC mass flow rate essentially halving as the starting TWC temperature increased from one experiment to the next. This was because that high HC emissions from an engine are also indicative of low combustion efficiency, much like the results for CO [28, 29]

The CO mass flow rate downstream of the TWC for the CDA strategy is presented in Fig. 4(b). In this case, the results are equivalent to those of the baseline for the first 130 s until the CDA period began. Once the CDA period began, there was then a change in the CO mass flow rate compared to the baseline that varied with the TWC starting temperature. When starting the engine with a TWC temperature of 100 °C or greater, the CDA period was able to reduce the duration taken for the TWC to reach light-off temperature for CO. However, for the ambient start, there was a large peak in CO mass flow rate of approx. 400 mg/s which is not shown on the graph to distinguish the other results. This peak was a result of the excess engine out CO mass flow rate whilst the TWC was not at a high enough temperature to fully convert it. Therefore, after the CDA period finishes the peak in CO mass flow rate is reduced. The change increase in engine-out CO mass flow rate was due to the two firing cylinders operating rich in order to maintain a total exhaust lambda of 1. Since the effect of rich combustion on CO emissions is the same as that for HC emissions there was also a peak in HC emissions during the CDA period for the ambient start as shown in Fig. 5(b).

The reduction in the time taken for the TWC to reach CO and HC light-off temperatures at starting catalyst temperatures of 100 °C or greater resulted from the exothermic reactions facilitated by the CDA operation. This also supports the reasoning behind the smaller increase in TWC temperature witnessed in Fig. 3 for the ambient TWC start compared to the higher temperature starts. Linking these results together, the higher CO and HC flow rates downstream of the TWC are species that were not oxidized over the TWC, so their heat was not added to the TWC, thus inhibiting the temperature rise of the TWC [30]. Comparing the total CO emissions allows for discussion regarding the applicability of the CDA strategy to improve vehicle emissions performance. Therefore, total cumulative mass of CO produced over the first 300 s of engine operation is presented in Table 2.

**Table 2:** Comparison of CO TWC light-off times and total mass over the first 300 s for each TWC starting temperature

TWC start temperature (°C)	Light-off time reduction (s)	Total CO mass baseline (g)	Total CO mass CDA (g)
amb.	0	7.08	10.18
100	60	5.49	3.99
150	31	2.82	2.32
200	30	1.14	0.65

These results demonstrate that the TWC temperature will be the determining factor as to how effective the CDA period will be in reducing the time required to reach CO light-off time. It could be possible that for engine starts from colder TWC start temperatures, the use of this CDA strategy could be useful if applied later in the warmup sequence when the TWC is within the appropriate temperature range. For these experimental conditions, it is suggested that once the TWC reaches a temperature of 181 °C (which was the temperature when the CDA period began for the 100 °C TWC start temperature) the CDA strategy will be most effective in CO reduction. Tuning the CDA period to begin at this catalyst temperature for each start could reduce total CO mass emissions for each engine start. The comparison of the CDA strategy to the baseline strategy in terms of time taken for the catalyst to reach HC light-off temperature and total HC mass emissions for each starting TWC temperature is shown in Table 3.

**Table 3:** Comparison of HC TWC light-off times and total mass over the first 300 s

TWC start temperature (°C)	Light-off time reduction (s)	Total HC mass baseline (g)	Total HC mass CDA (g)
amb.	0	2.95	2.87
100	61	1.87	1.49
150	44	1.11	1.02
200	34	0.48	0.39

**Fig. 6:**
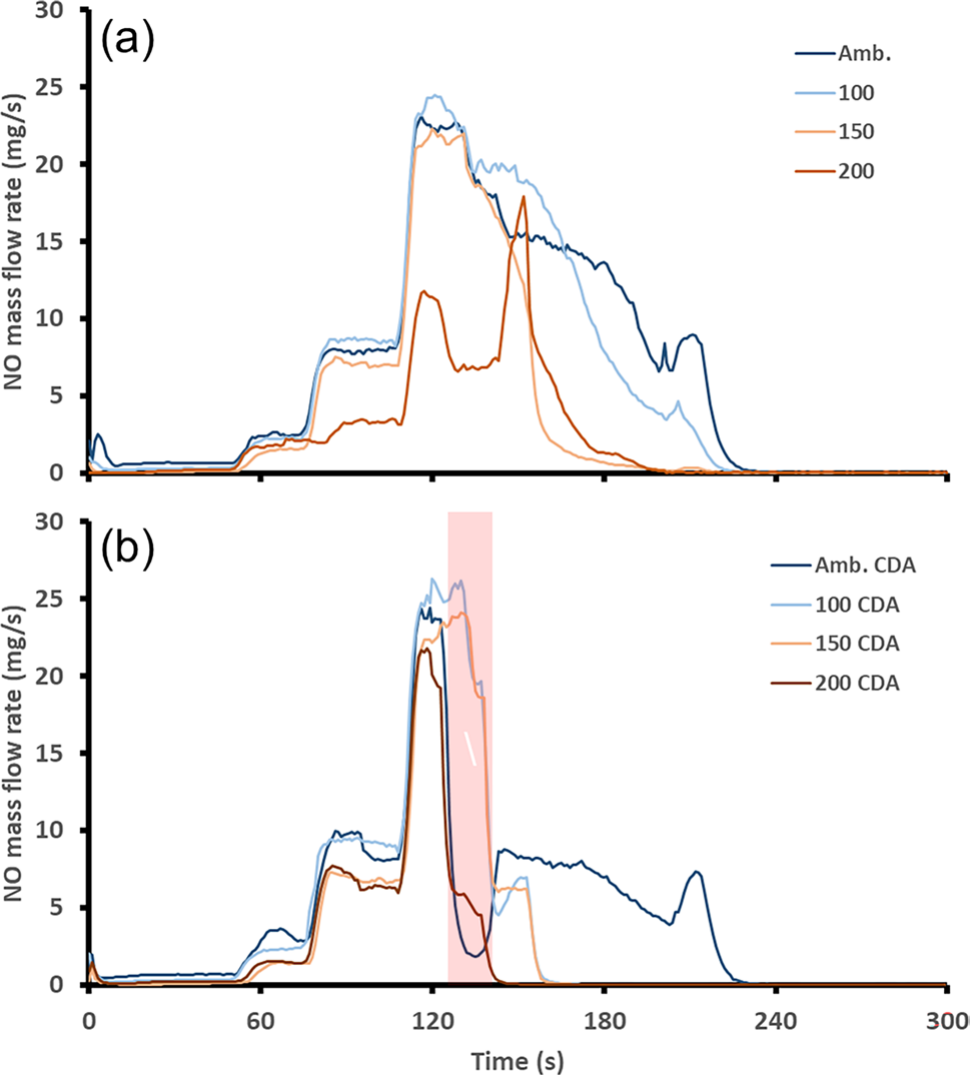
Post-TWC NO mass flow rate, **a** shows the baseline, **b** shows the start-up featuring the 15-second CDA period shown by the red shaded region

The cumulative HC emissions followed a similar trend to the CO. Although there was a different trend at the ambient start where the CDA strategy recorded an overall reduction, albeit a small one. Whilst there were similarities between emissions of CO and HCs at the ambient start in terms of the peak, the amplitude of the peak for the HC emissions was considerably smaller. There was also a notable reduction in the HC mass flow rate downstream of the TWC after the CDA period, although complete removal of HCs was still achieved at the same time as the baseline test. The later HC mass flow rate reduction after the CDA period offset the HC peak and therefore resulted in an overall reduction in HC mass emissions for the ambient start. As per the CO results, the CDA strategy achieved the greatest reduction in total emissions mass for the HCs when initiating the CDA period whilst the TWC was at a temperature of 181 °C. This would assist in the reduction of both CO and HCs as the optimization of the CDA strategy would be similar for both emissions species. The other regulated pollutant for vehicles is NO_*X*_. NO_*X*_ is the total emissions of NO and NO_2_. However, in this work only NO is presented as there were negligible emissions of NO_2_. This was because the SI engine was operated at stoichiometric for the vast majority of the testing limiting NO_2_ formation. The baseline NO mass flow rate downstream of the TWC is presented in Fig. 6(a).

The mass flow rate of NO during baseline operation was initially close to 0 mg/s. However, as the engine warmed up and load increased, the concentration of NO increased due to the dependence of NO formation (thermal NO) on combustion temperature [31]. In-cylinder NO formation started before the catalyst had reached the light-off temperature. Therefore, NO emissions associated with the start-up procedure contributed considerably to the total NO emissions during a vehicle drive cycle [32]. Each step increase in the NO mass flow rate was associated with an increase in the engine torque (Fig. 2). The ambient engine start produced the greatest mass flow rate of NO downstream of the TWC. This was due to the catalyst starting from the coldest temperature and therefore exhibiting low activity. For each of the baseline engine starts, as the starting TWC temperature increased so too did the performance of the TWC, thus resulting in reductions in NO mass flow rate. For all engine starts, there was a sharp drop in NO emissions during the CDA period due to the richer combustion in the two firing cylinders limiting NO production [33]. There was also a period of reduced tailpipe NO flow rate before light-off was achieved. This could be theorized as the oxygen storage capacity (OSC) being depleted during the rich CDA combustion period. There were reductions in light-off time for engine starts when the TWC started with a temperature greater than 100 °C. This was identified as the effect of two mechanisms. The first of which was the reduction in engine out NO mass flow rate during the CDA period with the second being the higher TWC activity post CDA period then maintaining a low NO flow rate. The effectiveness of the CDA strategy was reduced at the 200 °C TWC starting temperature where some NO reduction activity was already available due to the higher starting catalyst temperature. This led to a limit in the scope for improvement within the CDA period. It is anticipated that the adoption of this strategy for higher TWC start temperatures will continue to diminish the potential for light-off activity improvement as the TWC will already be operating at or close to 100% conversion efficiency. The accumulated NO mass emissions for each start temperature are shown in Table 4 for both the baseline and CDA experiments.

**Table 4:** Comparison of NO TWC light-off times and total mass over the first 300 s

TWC start temperature (°C)	Light-off time reduction (s)	Total NO mass baseline (g)	Total NO mass CDA (g)
amb.	0	1.94	1.30
100	59	1.78	1.13
150	31	1.13	0.94
200	47	0.70	0.61

Optimization of this CDA strategy to minimize NO emissions also requires consideration of the catalyst starting temperature. As per the results for CO and HCs, starting the CDA period with the TWC at a temperature of 181 °C would provide the greatest reduction in emissions performance. The effect of the CDA period on NO mass flow rate downstream of the catalyst, though it did improve at the ambient start. This contrasted with the CO and HC results. This was due to the inverse trend that during the CDA period, the NO emissions produced by the engine were reduced due to the rich operation. When looking to optimize the CDA strategy during an engine warm up procedure to reduce total regulated emissions, beginning the CDA period with the TWC temperature at 181 °C was the most effective for this work.

### Effect of Cylinder Deactivation on Nitrous Oxide Formation Over a TWC

N_2_O emissions form as a secondary emission over palladium based TWCs when conditions on the catalyst favor only the partial reduction of NO into N_2_O via reductants such as CO, H_2_ and HCs as shown in Eqs. (1), (2) and (3), respectively. The formation of N_2_O within the TWC typically occurs between 200 and 350 °C, although as catalysts age this window can increase [34].

1 $${\text{2NO + CO }} \to {\text{ N}}_{2} {\text{O}} + {\text{CO}}_{2}$$

2 $${\text{2NO}} + {\text{H}}_{2} \to {\text{ N}}_{2} {\text{O }} + {\text{ H}}_{2} {\text{O}}$$

3 $${\text{2NO}} + \frac{1}{9}{\text{C}}_{3} {\text{H}}_{6} \to {\text{ N}}_{2} {\text{O}} + \frac{1}{3}{\text{CO}}_{2} + \frac{1}{3}{\text{H}}_{2} {\text{O}}$$

**Fig. 7:**
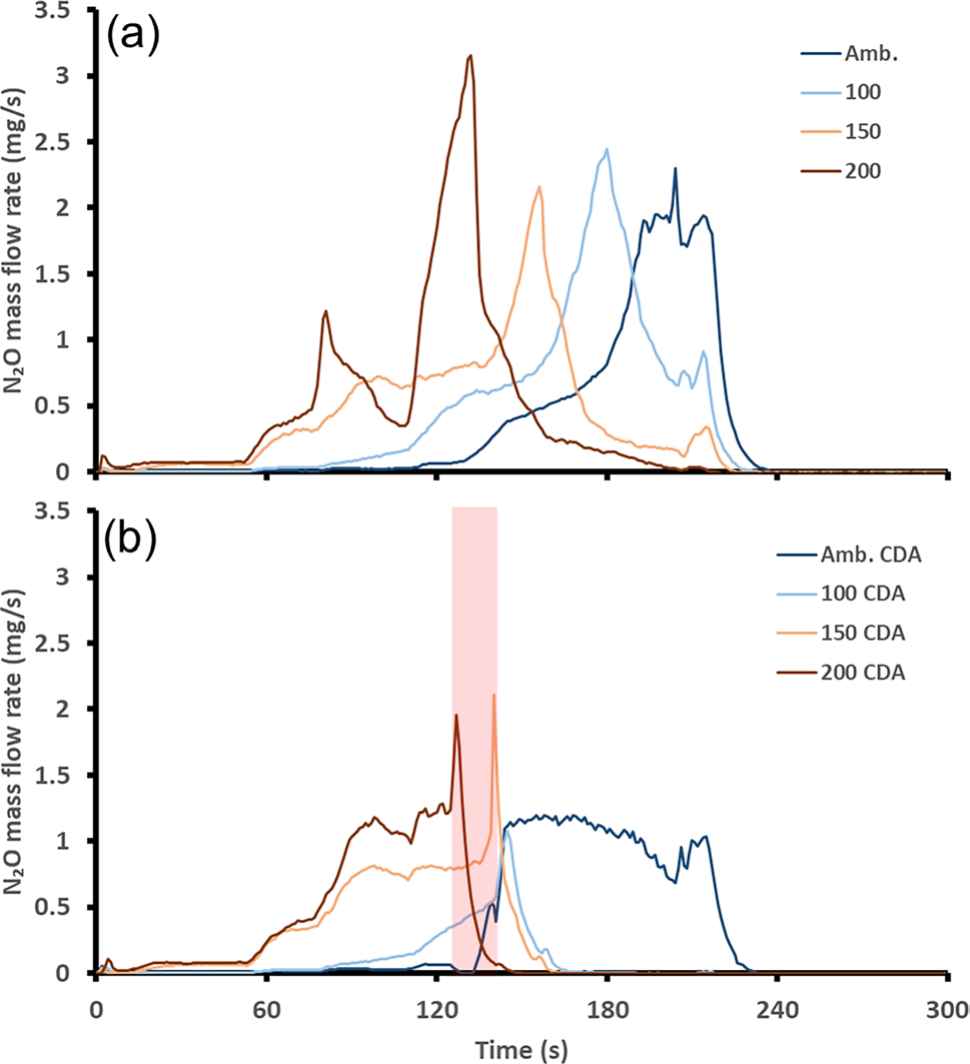
Post-TWC N_2_O mass flow rate, **a** shows the baseline, **b** shows the start-up featuring the 15-second CDA period shown by the red shaded region

Therefore, during TWC warm up when passing through this temperature range, N_2_O can be formed over the catalyst provided there are the required reactants available. For instance, if there is not any NO present to be reduced to N_2_O, then it will not form [35]. The N_2_O mass flow rate after the catalyst is plotted in Fig. 7 for both the baseline and the CDA start strategies.

The N_2_O formation peak occurred sooner as the TWC start temperature was increased for the baseline case. This is because the formation temperature region was reached earlier for the higher TWC temperature starts. The application of the CDA strategy reduced N_2_O emissions formation with respect to the baseline as well as changed the straightforward relationship between TWC starting temperature and N_2_O formation. For the ambient start, the formation of N_2_O was accelerated during the first 50 s after the CDA period as the TWC reached the formation region sooner. Following this, the formation of N_2_O reduced due to the TWC passing beyond the temperature region of N_2_O formation. Accelerating the time at which the TWC passes through, the N_2_O formation region was advantageous for this setup. This was because when the engine load increased later in the cycle, the increase in engine out NO associated with this was not converted into N_2_O, as full NO reduction was taking place due to the TWC operating at a temperature greater than the N_2_O formation region. Furthermore, due to the rich operation of the firing cylinders during the CDA period, the reduction in engine out NO would have also inhibited the formation of N_2_O. When starting the engine with a TWC of 100 °C or greater, there was a marked reduction in N_2_O formation over the TWC. This most likely occurred due to the rapid and large temperature increase (Fig. 3) from 200 °C up to 400 °C due to the CDA operation preventing the TWC from operating at temperatures favorable for N_2_O formation. This proves that the application of this CDA method could be an effective strategy to reduce N_2_O formation during catalyst warm up events. Table 5 shows the N_2_O comparison between each strategy over the first 5 min of engine operation.

**Table 5:** Comparison of time spent in N_2_O formation region and total mass over the first 300 s

TWC start temperature (°C)	Total N_2_O mass baseline (g)	Total N_2_O mass CDA (g)
amb.	0.10	0.09
100	0.11	0.02
150	0.10	0.06
200	0.11	0.07

Based on the total cumulative N_2_O emissions, it is suggested that the CDA period should be initiated when the TWC temperature reaches 181 °C as this achieved the greatest reduction in N_2_O. This is also a comparable temperature to the most effective for regulated emissions reduction in this study. When starting the CDA period whilst the TWC is at 181 °C, it means that the CDA period starts just before the TWC enters the temperature range favorable for N_2_O formation. During the CDA period, the reduced engine out NO and increased TWC temperature would limit the formation of N_2_O. Following the CDA period, the TWC temperature would be maintained above the temperature formation region for N_2_O therefore limiting N_2_O emissions.

### Effect of Cylinder Deactivation on Ammonia Formation Over a TWC

Similar in nature to the formation of N_2_O, the formation of NH_3_ as a secondary emission has been reported to occur over palladium based TWCs. The formation window for NH_3_ occurs at a higher temperature of between 300 and 450 °C with rich conditions exacerbating NH_3_ formation [36]. The formation of NH_3_ takes place through the process of isocyanate (HNCO) hydrolysis which also requires the presence of NO [37] as shown in Eqs. (4) and (5).

4 $${\text{2NO + 4CO + 2H}}_{2} {\text{O}} + {\text{H}}_{2} \to {\text{ 2NH}}_{3} + {\text{ 4CO}}_{2}$$

5 $${\text{NH}}_{3} {\text{ + 2NO + 5CO + 3H}}_{2} {\text{O}} \to {\text{3NH}}_{3} + {\text{5CO}}_{2}$$

**Fig. 8:**
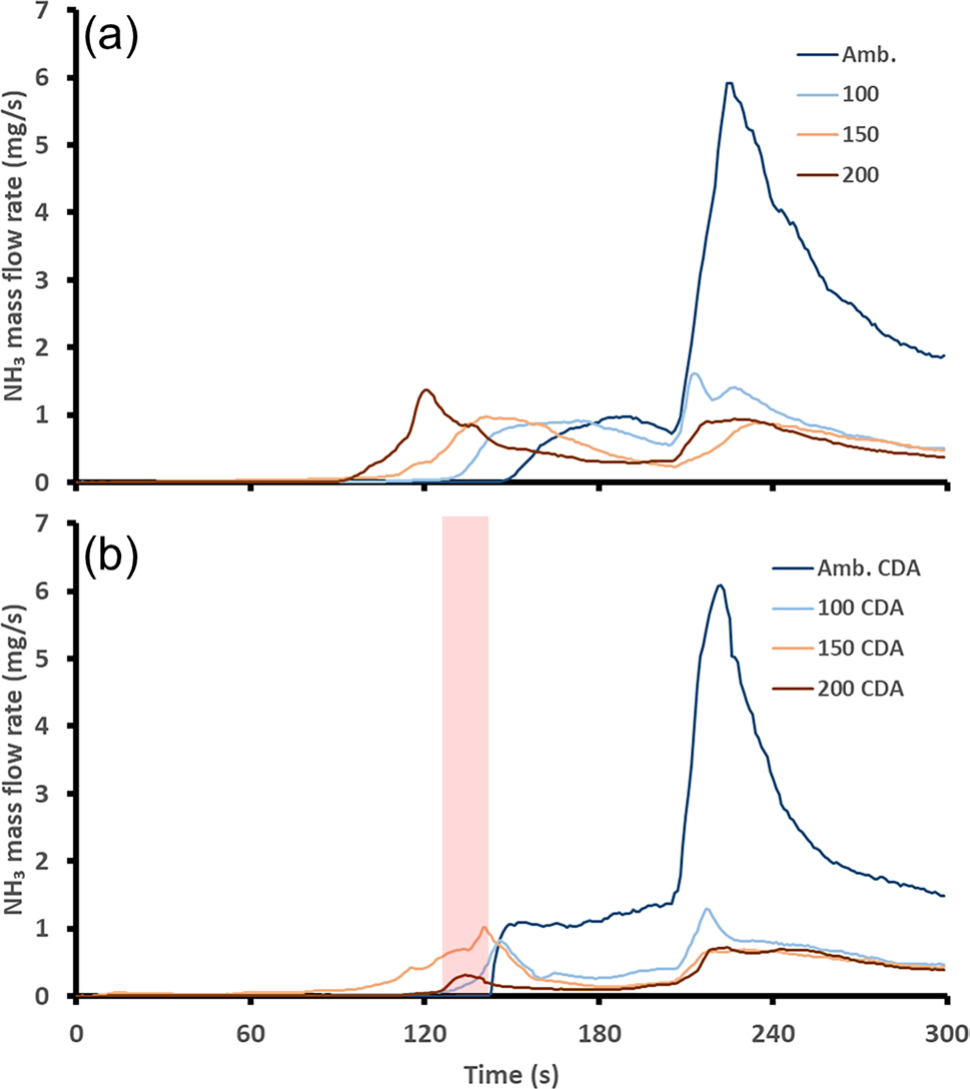
Post-TWC NH_3_ mass flow rate, **a** shows the baseline, **b** shows the start-up featuring the 15-second CDA period shown by the red shaded region

Equation (4) shows the overall reaction pathway for the formation of NH_3_ from NO, CO and H_2_. This occurs via the formation of HNCO as the intermediate from these three species before its subsequent hydrolysis with H_2_O to produce NH_3_. Equation 5 shows the same reaction pathway but this time H_2_ is replaced with any NH_3_ that is present. Since this second pathway produces 3 mol of NH_3_ for every mole of NH_3_ on the reactants side, it is an amplifying pathway that can increase the amount of NH_3_ threefold [37]. The mass flow rate of NH_3_ for each catalyst start temperature is shown in Fig. 8. Similar to the peaks that occurred in the N_2_O mass flow rate the peaks in NH_3_ formation are linked to increases in engine out NO and the TWC temperature.

The largest peak in NH_3_ emissions was observed during the ambient engine start. This occurred as the engine out NO flow rate increased due to the engine load increase from 30 to 60 N m. The implementation of CDA for the ambient start had little impact on the NH_3_ mass flow rate. There was a small increase in the amount of NH_3_ formed over the TWC before the peak in NH_3_ flow rate that occurred as the engine load increased to 60 N m. This was a result of the small increase in TWC temperature that resulted from the CDA period. There was the benefit later of a shortened period where the TWC temperature was within the NH_3_ formation window shown by the narrower peak in NH_3_ flow rate. The net effect of both of these periods was to achieve a very similar total mass of NH_3_ formed during the warmup period. This ambient experiment shows that it is possible that applying the CDA period before the TWC is close to light-off could be detrimental and push the TWC into the NH_3_ formation region. For engine starts with a TWC temperature of 100 °C or greater, there was a reduction in the mass flow rate of NH_3_ after the CDA period compared to the baseline. This occurred as a result of the large drop in engine out NO emissions during the CDA period due to the rich operation of the firing cylinders. This reduced the NO availability for HNCO formation. There was also an extended period of reduced NH_3_ formation that continued after the CDA period. This was likely a result of increasing the TWC temperature beyond the NH_3_ formation region and filling of the OSC on the catalyst due to the excess oxygen pumped to the TWC by the deactivated cylinder [38]. This was the case even though the two firing cylinders were operating under rich conditions. Leaner conditions over a palladium-based catalyst are widely known to inhibit NH_3_ formation [36]. The total NH_3_ mass flow rates for both start strategies at each TWC start temperature are presented in Table 6. The light-off time reduction was not given since NH_3_ was produced rather than converted over the catalyst.

**Table 6:** Comparison of the total NH_3_ accumulated mass over the whole start-up experimental sequences

TWC start temperature (°C)	Total NH_3_ mass baseline (g)	Total NH_3_ mass CDA (g)
amb.	0.34	0.34
100	0.14	0.10
150	0.12	0.10
200	0.09	0.06

The impact of this CDA strategy on NH_3_ emissions at different TWC start temperatures was positive for all starting temperatures other than the ambient start. The optimization of using CDA to reduce the formation of NH_3_ over the TWC during PHEV operation hinges on the timing at which the CDA period is activated. This was evident with the ambient start where the application of the CDA strategy pushed the TWC temperature up into the window of NH_3_ formation. If a controller was optimized to use the CDA strategy, it would need to balance the emissions benefit of achieving TWC light-off sooner without applying the TWC heating strategy too early and risking increased NH_3_ formation.

### Effect of Cylinder Deactivation on Fuel Consumption

Due to the changes in engine operation and combustion process, the brake thermal efficiency and therefore fuel consumption were both affected. This is standard for all vehicle catalyst heating strategies since the energy to heat the catalyst must come from somewhere. The total fuel consumed during each of the engine starts is shown in Table 7.

**Table 7:** Comparison of the total fuel consumption over the whole start-up experimental sequences

TWC start temperature (°C)	Total fuel consumption baseline (g)	Total fuel consumption CDA (g)
amb.	211.4	213.8
100	194.8	195.9
150	191.0	193.6
200	190.6	191.5

The first trend observed when looking at efficiency for each experiment was the reduced fuel consumption for each catalyst warm up period as the starting temperature increased. This was a result of improved mixture formation and combustion within the cylinder at higher temperatures and improved mechanical efficiency at higher engine temperatures. This trend was true for both the baseline experiments and the experiments that employed the CDA strategy. There was a trend in the change in fuel consumption observed between the baseline experiments and the CDA experiments. This showed that there was a consistent fuel consumption penalty for the use of this TWC heating strategy at all temperatures. However, the differences in fuel consumption for each starting temperature are all between 0.5 and 1.4% over the 300-second duration recorded here. Other catalyst heating strategies reported overall increases in fuel consumption throughout a full drive cycle of between 0.5 and 2% [11, 12]. If the CDA strategy was employed over a full drive cycle, the total increase in fuel consumption would reduce as the CDA period would represent an overall lower percentage of the whole journey time. This suggests it is an effective energy efficient solution. Further to this, over a full drive cycle, it would be possible to optimize the CDA strategy as part of an intelligent control strategy [39]. This strategy could adjust CDA duration, and timings based on catalyst temperature such that it only occurs within the optimum window for maximum emissions reduction with minimal fuel consumption penalty.

## Conclusions

This work provides new understanding and experimental evidence of a novel energy efficient catalyst heating strategy for primary and secondary emissions control from PHEVs. The strategy, which is based on engine cylinder deactivation, has been studied at several catalyst monolith starting temperatures mimicking the catalyst conditions seen in PHEVs and HEVs as a result of the intermittent engine operation over each single journey.

This work provides the opportunity to achieve significant reductions in the time taken to reach catalyst light-off temperature for catalyst starting temperatures between 100 and 200 °C. The maximum reductions in catalyst light-off time were achieved at the 100 °C starting catalyst temperature where light-off time was reduced by approximately 60 s. Yet on the other hand, if the catalyst starting temperature was too high then the benefit of the proposed strategy would be diminished. Reductions in both primary and secondary N_2_O and NH_3_ tailpipe emissions were also achieved simultaneously using the proposed strategy.

In order to further implement this strategy, the cylinder deactivation timings and duration should be optimized for each temperature. The application of these strategies on HEVs and PHEVs over Real Driving Emissions would reveal the full emissions-reducing potential alongside any fuel consumption impact.

## Abbreviations

CDA: Cylinder deactivation
DSF: Dynamic skip fire
EIMS: Electron impact mass spectrometer
EMS: Energy management strategy
FTIR: Fourier transform infrared
GDI: Gasoline direct injection
HC: Hydrocarbon
HEV: Hybrid electric vehicle
ICE: Internal combustion engine
PFI: Port fuel injection
PGM: Platinum group metal
SI: Spark ignition
TWC: Three-way catalyst
